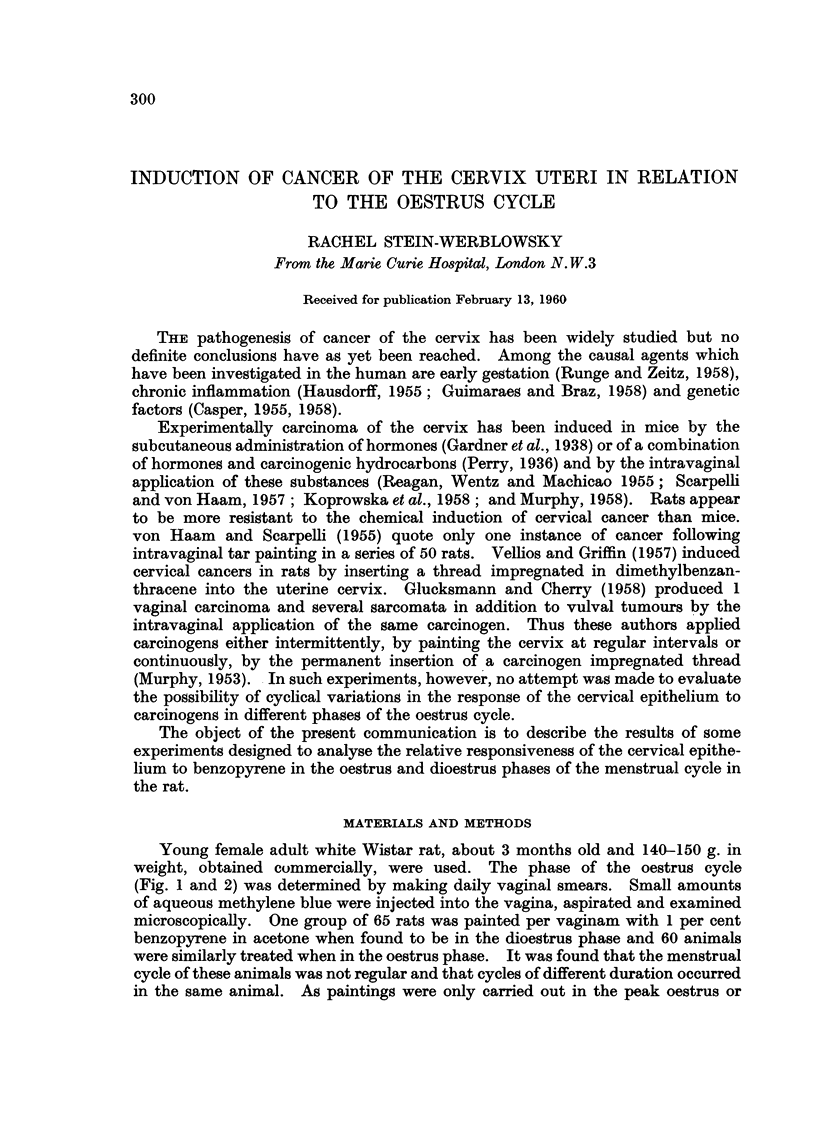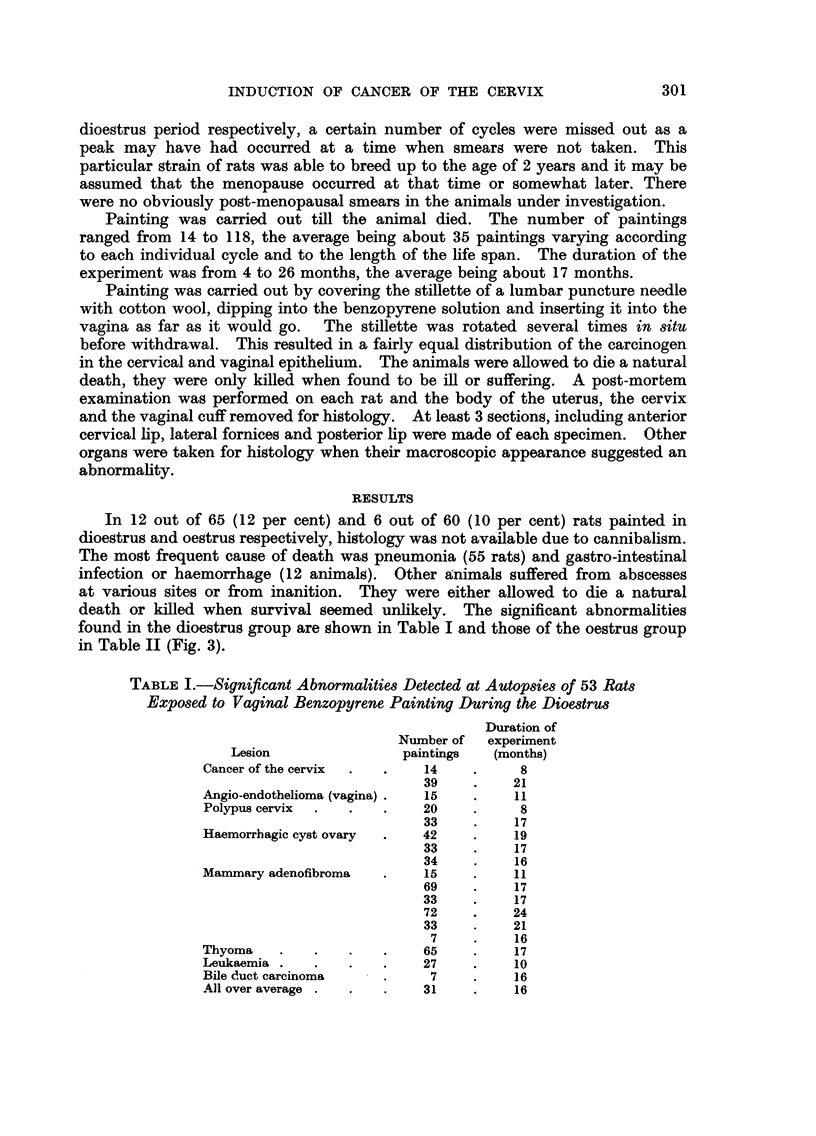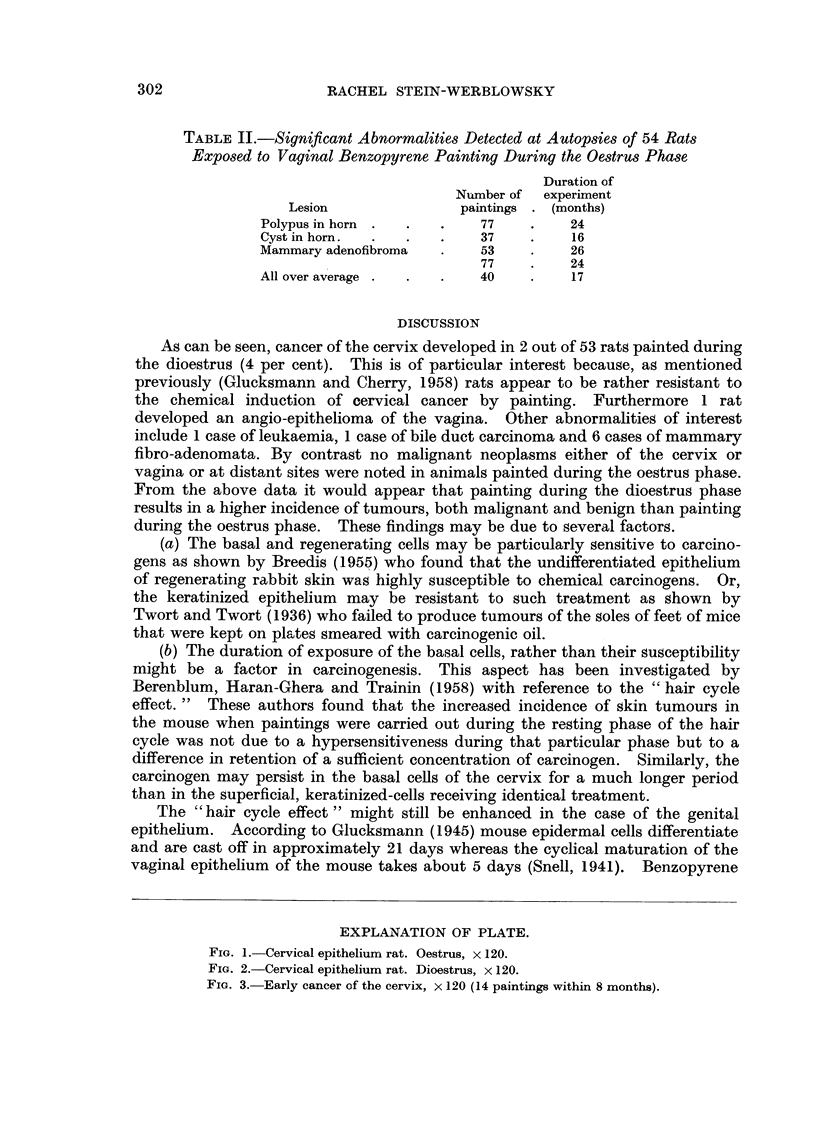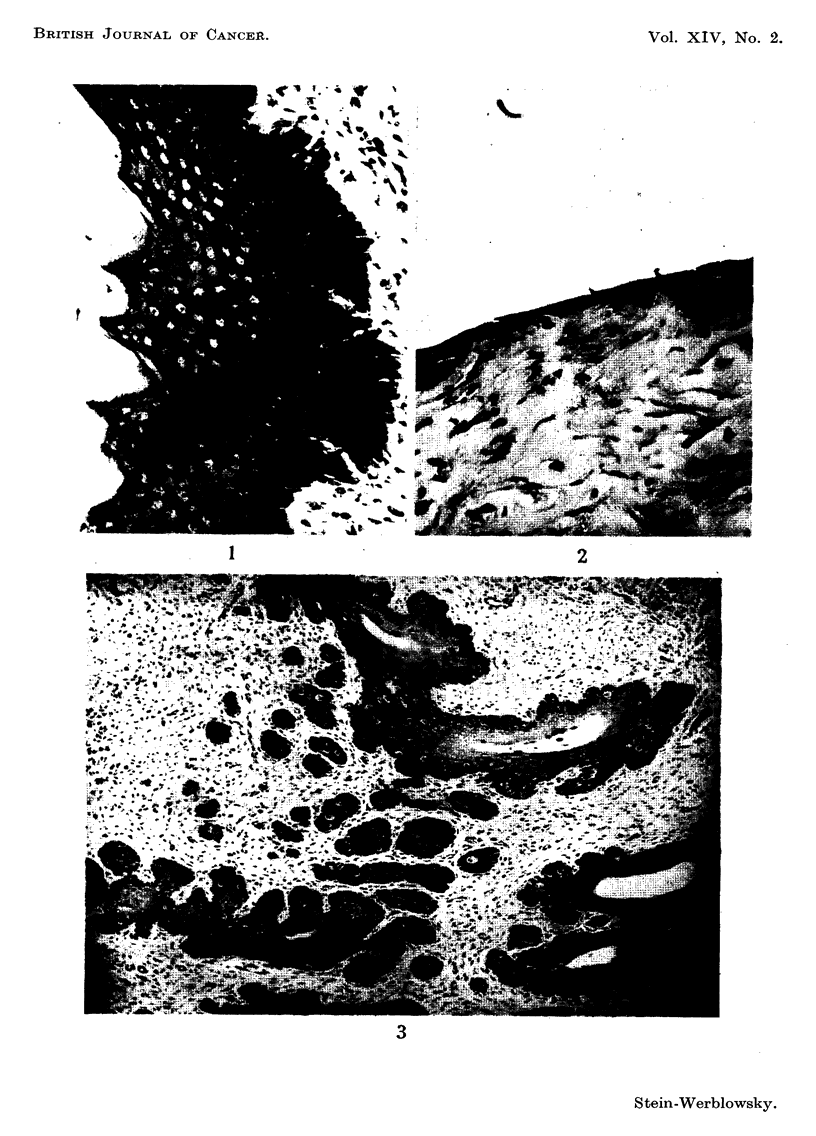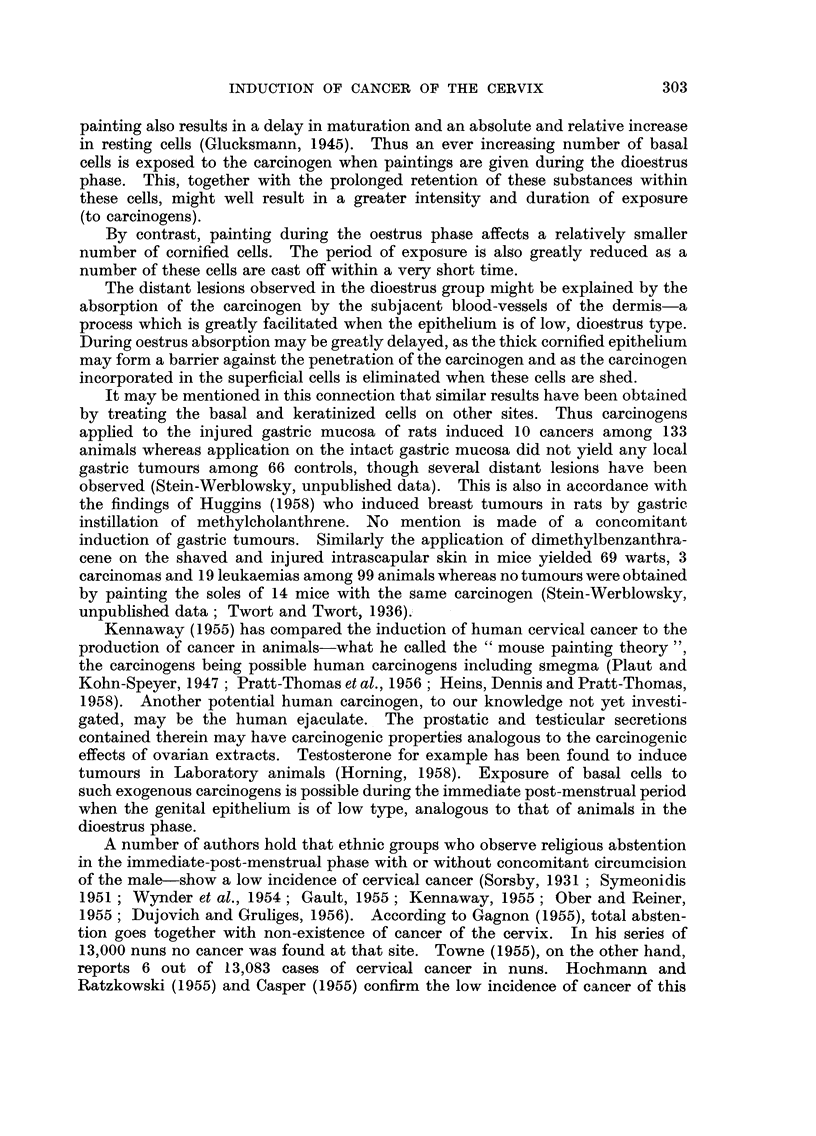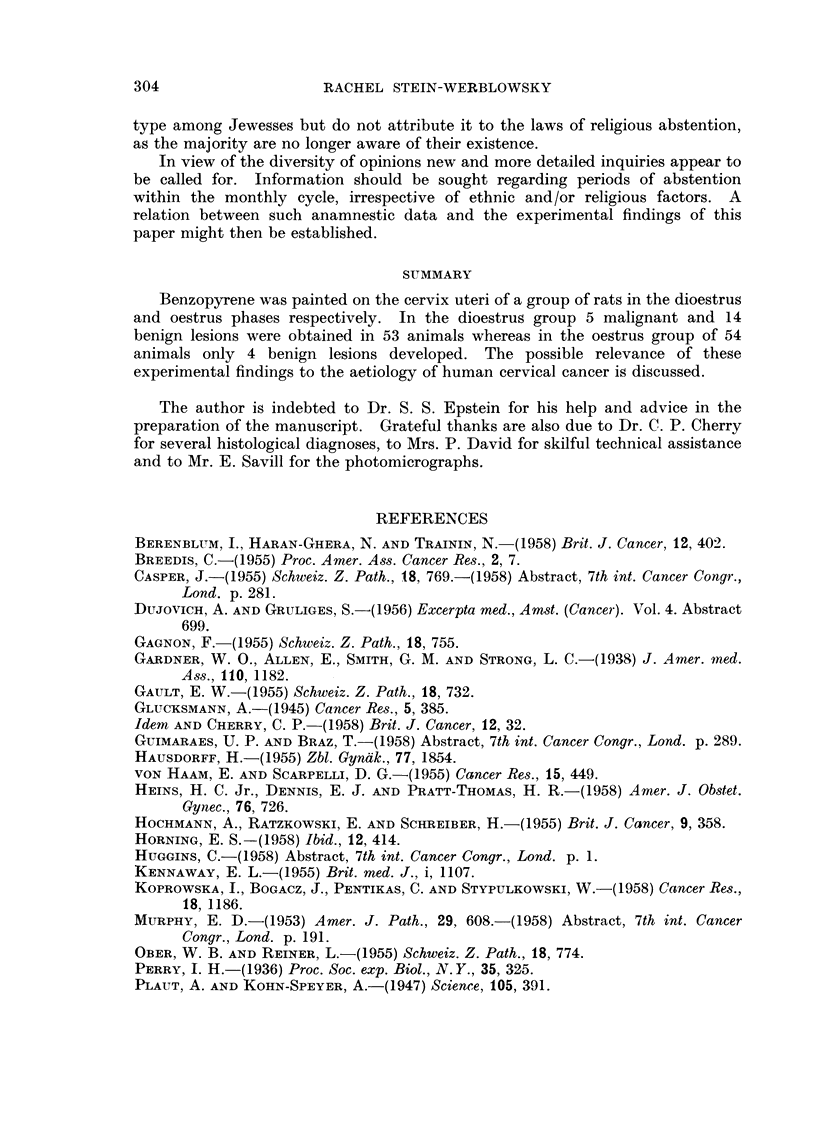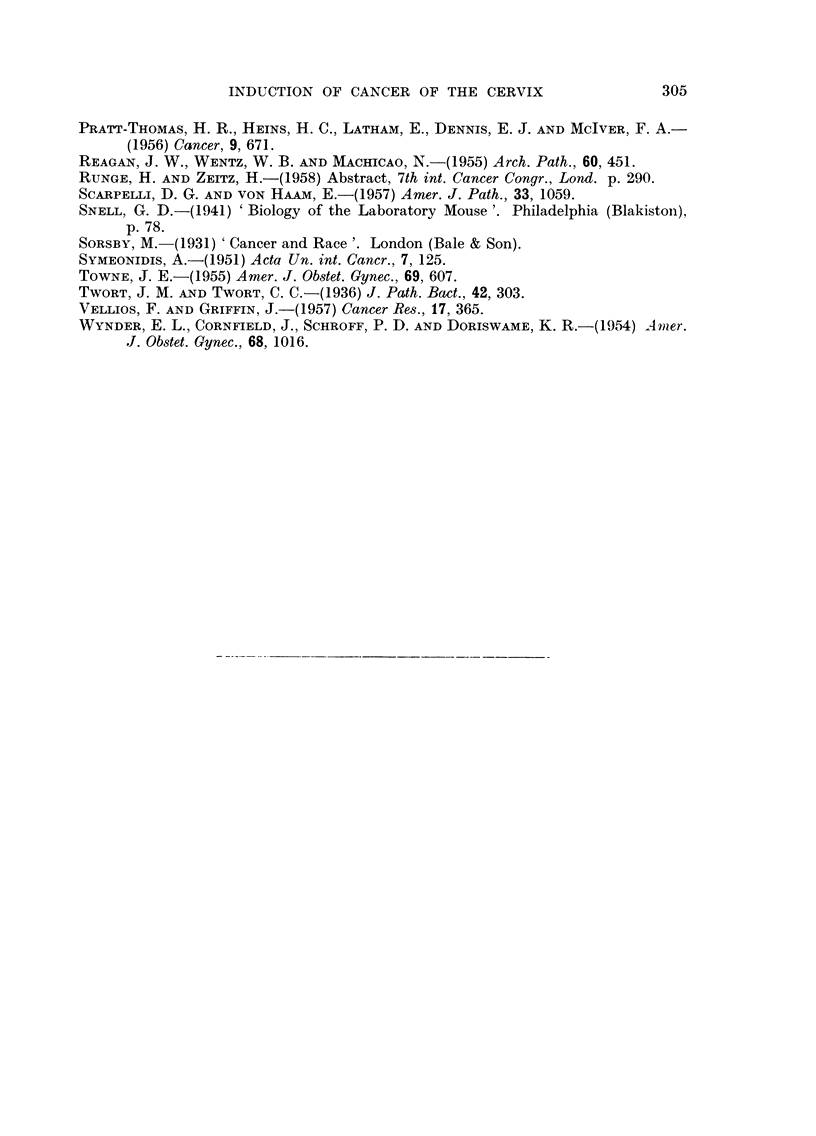# Induction of Cancer of the Cervix Uteri in Relation to the Oestrus Cycle

**DOI:** 10.1038/bjc.1960.35

**Published:** 1960-06

**Authors:** Rachel Stein-Werblowsky

## Abstract

**Images:**


### INDUCTION OF CANCER OF THE CERVIX UTERI IN RELATION TO THE OESTRUS CYCLE

RACHEL STEIN-WERBLOWSKY
*From the Marie Curie Hospital, London N.W.3*

Received for publication February 13, 1960

THE pathogenesis of cancer of the cervix has been widely studied but no definite conclusions have as yet been reached. Among the causal agents which have been investigated in the human are early gestation (Runge and Zeitz, 1958), chronic inflammation (Hausdorff, 1955 ; Guimaraes and Braz, 1958) and genetic factors (Casper, 1955, 1958).

Experimentally carcinoma of the cervix has been induced in mice by the subcutaneous administration of hormones (Gardner *et al.*, 1938) or of a combination of hormones and carcinogenic hydrocarbons (Perry, 1936) and by the intravaginal application of these substances (Reagan, Wentz and Machicao 1955 ; Scarpelli and von Haam, 1957 ; Koprowska *et al.*, 1958 ; and Murphy, 1958). Rats appear to be more resistant to the chemical induction of cervical cancer than mice. von Haam and Scarpelli (1955) quote only one instance of cancer following intravaginal tar painting in a series of 50 rats. Vellios and Griffin (1957) induced cervical cancers in rats by inserting a thread impregnated in dimethylbenzanthracene into the uterine cervix. Glucksmann and Cherry (1958) produced 1 vaginal carcinoma and several sarcomata in addition to vulval tumours by the intravaginal application of the same carcinogen. Thus these authors applied carcinogens either intermittently, by painting the cervix at regular intervals or continuously, by the permanent insertion of a carcinogen impregnated thread (Murphy, 1953). In such experiments, however, no attempt was made to evaluate the possibility of cyclical variations in the response of the cervical epithelium to carcinogens in different phases of the oestrus cycle.

The object of the present communication is to describe the results of some experiments designed to analyse the relative responsiveness of the cervical epithelium to benzopyrene in the oestrus and dioestrus phases of the menstrual cycle in the rat.

### MATERIALS AND METHODS

Young female adult white Wistar rat, about 3 months old and 140–150 g. in weight, obtained commercially, were used. The phase of the oestrus cycle (Fig. 1 and 2) was determined by making daily vaginal smears. Small amounts of aqueous methylene blue were injected into the vagina, aspirated and examined microscopically. One group of 65 rats was painted per vaginam with 1 per cent benzopyrene in acetone when found to be in the dioestrus phase and 60 animals were similarly treated when in the oestrus phase. It was found that the menstrual cycle of these animals was not regular and that cycles of different duration occurred in the same animal. As paintings were only carried out in the peak oestrus or dioestrus period respectively, a certain number of cycles were missed out as a peak may have had occurred at a time when smears were not taken. This particular strain of rats was able to breed up to the age of 2 years and it may be assumed that the menopause occurred at that time or somewhat later. There were no obviously post-menopausal smears in the animals under investigation.

Painting was carried out till the animal died. The number of paintings ranged from 14 to 118, the average being about 35 paintings varying according to each individual cycle and to the length of the life span. The duration of the experiment was from 4 to 26 months, the average being about 17 months.

Painting was carried out by covering the stillette of a lumbar puncture needle with cotton wool, dipping into the benzopyrene solution and inserting it into the vagina as far as it would go. The stillette was rotated several times *in situ* before withdrawal. This resulted in a fairly equal distribution of the carcinogen in the cervical and vaginal epithelium. The animals were allowed to die a natural death, they were only killed when found to be ill or suffering. A post-mortem examination was performed on each rat and the body of the uterus, the cervix and the vaginal cuff removed for histology. At least 3 sections, including anterior cervical lip, lateral fornices and posterior lip were made of each specimen. Other organs were taken for histology when their macroscopic appearance suggested an abnormality.

### RESULTS

In 12 out of 65 (12 per cent) and 6 out of 60 (10 per cent) rats painted in dioestrus and oestrus respectively, histology was not available due to cannibalism. The most frequent cause of death was pneumonia (55 rats) and gastro-intestinal infection or haemorrhage (12 animals). Other animals suffered from abscesses at various sites or from inanition. They were either allowed to die a natural death or killed when survival seemed unlikely. The significant abnormalities found in the dioestrus group are shown in Table I and those of the oestrus group in Table II (Fig. 3).

TABLE I.—*Significant Abnormalities Detected at Autopsies of* 53 *Rats Exposed to Vaginal Benzopyrene Painting During the Dioestrus*

| Lesion | Number of paintings | Duration of experiment (months) |
|---|---|---|
| Cancer of the cervix | 14 | 8 |
| | 39 | 21 |
| Angio-endothelioma (vagina) | 15 | 11 |
| Polypus cervix | 20 | 8 |
| | 33 | 17 |
| Haemorrhagic cyst ovary | 42 | 19 |
| | 33 | 17 |
| | 34 | 16 |
| Mammary adenofibroma | 15 | 11 |
| | 69 | 17 |
| | 33 | 17 |
| | 72 | 24 |
| | 33 | 21 |
| | 7 | 16 |
| Thyoma | 65 | 17 |
| Leukaemia | 27 | 10 |
| Bile duct carcinoma | 7 | 16 |
| All over average | 31 | 16 |

TABLE II.—*Significant Abnormalities Detected at Autopsies of 54 Rats Exposed to Vaginal Benzopyrene Painting During the Oestrus Phase*

| Lesion | Number of paintings | Duration of experiment (months) |
|---|---|---|
| Polypus in horn | 77 | 24 |
| Cyst in horn | 37 | 16 |
| Mammary adenofibroma | 53 | 26 |
| | 77 | 24 |
| All over average | 40 | 17 |

### DISCUSSION

As can be seen, cancer of the cervix developed in 2 out of 53 rats painted during the dioestrus (4 per cent). This is of particular interest because, as mentioned previously (Glucksmann and Cherry, 1958) rats appear to be rather resistant to the chemical induction of cervical cancer by painting. Furthermore 1 rat developed an angio-epithelioma of the vagina. Other abnormalities of interest include 1 case of leukaemia, 1 case of bile duct carcinoma and 6 cases of mammary fibro-adenomata. By contrast no malignant neoplasms either of the cervix or vagina or at distant sites were noted in animals painted during the oestrus phase. From the above data it would appear that painting during the dioestrus phase results in a higher incidence of tumours, both malignant and benign than painting during the oestrus phase. These findings may be due to several factors.

(*a*) The basal and regenerating cells may be particularly sensitive to carcinogens as shown by Breedis (1955) who found that the undifferentiated epithelium of regenerating rabbit skin was highly susceptible to chemical carcinogens. Or, the keratinized epithelium may be resistant to such treatment as shown by Twort and Twort (1936) who failed to produce tumours of the soles of feet of mice that were kept on plates smeared with carcinogenic oil.

(*b*) The duration of exposure of the basal cells, rather than their susceptibility might be a factor in carcinogenesis. This aspect has been investigated by Berenblum, Haran-Ghera and Trainin (1958) with reference to the "hair cycle effect." These authors found that the increased incidence of skin tumours in the mouse when paintings were carried out during the resting phase of the hair cycle was not due to a hypersensitiveness during that particular phase but to a difference in retention of a sufficient concentration of carcinogen. Similarly, the carcinogen may persist in the basal cells of the cervix for a much longer period than in the superficial, keratinized-cells receiving identical treatment.

The "hair cycle effect" might still be enhanced in the case of the genital epithelium. According to Glucksmann (1945) mouse epidermal cells differentiate and are cast off in approximately 21 days whereas the cyclical maturation of the vaginal epithelium of the mouse takes about 5 days (Snell, 1941). Benzopyrene

---

EXPLANATION OF PLATE.

FIG. 1.—Cervical epithelium rat. Oestrus, ×120.

FIG. 2.—Cervical epithelium rat. Dioestrus, ×120.

FIG. 3.—Early cancer of the cervix, ×120 (14 paintings within 8 months).

1

2

3

Stein-Werblowsky.

painting also results in a delay in maturation and an absolute and relative increase in resting cells (Glucksmann, 1945). Thus an ever increasing number of basal cells is exposed to the carcinogen when paintings are given during the dioestrus phase. This, together with the prolonged retention of these substances within these cells, might well result in a greater intensity and duration of exposure (to carcinogens).

By contrast, painting during the oestrus phase affects a relatively smaller number of cornified cells. The period of exposure is also greatly reduced as a number of these cells are cast off within a very short time.

The distant lesions observed in the dioestrus group might be explained by the absorption of the carcinogen by the subjacent blood-vessels of the dermis—a process which is greatly facilitated when the epithelium is of low, dioestrus type. During oestrus absorption may be greatly delayed, as the thick cornified epithelium may form a barrier against the penetration of the carcinogen and as the carcinogen incorporated in the superficial cells is eliminated when these cells are shed.

It may be mentioned in this connection that similar results have been obtained by treating the basal and keratinized cells on other sites. Thus carcinogens applied to the injured gastric mucosa of rats induced 10 cancers among 133 animals whereas application on the intact gastric mucosa did not yield any local gastric tumours among 66 controls, though several distant lesions have been observed (Stein-Werblowsky, unpublished data). This is also in accordance with the findings of Huggins (1958) who induced breast tumours in rats by gastric instillation of methylcholanthrene. No mention is made of a concomitant induction of gastric tumours. Similarly the application of dimethylbenzanthracene on the shaved and injured intrascapular skin in mice yielded 69 warts, 3 carcinomas and 19 leukaemias among 99 animals whereas no tumours were obtained by painting the soles of 14 mice with the same carcinogen (Stein-Werblowsky, unpublished data ; Twort and Twort, 1936).

Kennaway (1955) has compared the induction of human cervical cancer to the production of cancer in animals—what he called the " mouse painting theory ", the carcinogens being possible human carcinogens including smegma (Plaut and Kohn-Speyer, 1947 ; Pratt-Thomas *et al.*, 1956 ; Heins, Dennis and Pratt-Thomas, 1958). Another potential human carcinogen, to our knowledge not yet investigated, may be the human ejaculate. The prostatic and testicular secretions contained therein may have carcinogenic properties analogous to the carcinogenic effects of ovarian extracts. Testosterone for example has been found to induce tumours in Laboratory animals (Horning, 1958). Exposure of basal cells to such exogenous carcinogens is possible during the immediate post-menstrual period when the genital epithelium is of low type, analogous to that of animals in the dioestrus phase.

A number of authors hold that ethnic groups who observe religious abstention in the immediate-post-menstrual phase with or without concomitant circumcision of the male—show a low incidence of cervical cancer (Sorsby, 1931 ; Symeonidis 1951 ; Wynder *et al.*, 1954 ; Gault, 1955 ; Kennaway, 1955 ; Ober and Reiner, 1955 ; Dujovich and Gruliges, 1956). According to Gagnon (1955), total abstention goes together with non-existence of cancer of the cervix. In his series of 13,000 nuns no cancer was found at that site. Towne (1955), on the other hand, reports 6 out of 13,083 cases of cervical cancer in nuns. Hochmann and Ratzkowski (1955) and Casper (1955) confirm the low incidence of cancer of this type among Jewesses but do not attribute it to the laws of religious abstention, as the majority are no longer aware of their existence.

In view of the diversity of opinions new and more detailed inquiries appear to be called for. Information should be sought regarding periods of abstention within the monthly cycle, irrespective of ethnic and/or religious factors. A relation between such anamnestic data and the experimental findings of this paper might then be established.

### SUMMARY

Benzopyrene was painted on the cervix uteri of a group of rats in the dioestrus and oestrus phases respectively. In the dioestrus group 5 malignant and 14 benign lesions were obtained in 53 animals whereas in the oestrus group of 54 animals only 4 benign lesions developed. The possible relevance of these experimental findings to the aetiology of human cervical cancer is discussed.

The author is indebted to Dr. S. S. Epstein for his help and advice in the preparation of the manuscript. Grateful thanks are also due to Dr. C. P. Cherry for several histological diagnoses, to Mrs. P. David for skilful technical assistance and to Mr. E. Savill for the photomicrographs.

### REFERENCES

Berenblum, I., Haran-Ghera, N. and Trainin, N.—(1958) *Brit. J. Cancer*, **12**, 402.
Breedis, C.—(1955) *Proc. Amer. Ass. Cancer Res.*, **2**, 7.
Casper, J.—(1955) *Schweiz. Z. Path.*, **18**, 769.—(1958) Abstract, *7th int. Cancer Congr., Lond.* p. 281.
Dujovich, A. and Gruliges, S.—(1956) *Excerpta med., Amst.* (*Cancer*). Vol. 4. Abstract 699.
Gagnon, F.—(1955) *Schweiz. Z. Path.*, **18**, 755.
Gardner, W. O., Allen, E., Smith, G. M. and Strong, L. C.—(1938) *J. Amer. med. Ass.*, **110**, 1182.
Gault, E. W.—(1955) *Schweiz. Z. Path.*, **18**, 732.
Glucksmann, A.—(1945) *Cancer Res.*, **5**, 385.
*Idem* and Cherry, C. P.—(1958) *Brit. J. Cancer*, **12**, 32.
Guimaraes, U. P. and Braz, T.—(1958) Abstract, *7th int. Cancer Congr., Lond.* p. 289.
Hausdorff, H.—(1955) *Zbl. Gynäk.*, **77**, 1854.
von Haam, E. and Scarpelli, D. G.—(1955) *Cancer Res.*, **15**, 449.
Heins, H. C. Jr., Dennis, E. J. and Pratt-Thomas, H. R.—(1958) *Amer. J. Obstet. Gynec.*, **76**, 726.
Hochmann, A., Ratzkowski, E. and Schreiber, H.—(1955) *Brit. J. Cancer*, **9**, 358.
Horning, E. S.—(1958) *Ibid.*, **12**, 414.
Huggins, C.—(1958) Abstract, *7th int. Cancer Congr., Lond.* p. 1.
Kennaway, E. L.—(1955) *Brit. med. J.*, i, 1107.
Koprowska, I., Bogacz, J., Pentikas, C. and Stypulkowski, W.—(1958) *Cancer Res.*, **18**, 1186.
Murphy, E. D.—(1953) *Amer. J. Path.*, **29**, 608.—(1958) Abstract, *7th int. Cancer Congr., Lond.* p. 191.
Ober, W. B. and Reiner, L.—(1955) *Schweiz. Z. Path.*, **18**, 774.
Perry, I. H.—(1936) *Proc. Soc. exp. Biol., N.Y.*, **35**, 325.
Plaut, A. and Kohn-Speyer, A.—(1947) *Science*, **105**, 391.

Pratt-Thomas, H. R., Heins, H. C., Latham, E., Dennis, E. J. and McIver, F. A.—(1956) *Cancer*, **9**, 671.

Reagan, J. W., Wentz, W. B. and Machicao, N.—(1955) *Arch. Path.*, **60**, 451.

Runge, H. and Zeitz, H.—(1958) Abstract, *7th int. Cancer Congr., Lond.* p. 290.

Scarpelli, D. G. and von Haam, E.—(1957) *Amer. J. Path.*, **33**, 1059.

Snell, G. D.—(1941) ' Biology of the Laboratory Mouse '. Philadelphia (Blakiston), p. 78.

Sorsby, M.—(1931) ' Cancer and Race '. London (Bale & Son).

Symeonidis, A.—(1951) *Acta Un. int. Cancr.*, **7**, 125.

Towne, J. E.—(1955) *Amer. J. Obstet. Gynec.*, **69**, 607.

Twort, J. M. and Twort, C. C.—(1936) *J. Path. Bact.*, **42**, 303.

Vellios, F. and Griffin, J.—(1957) *Cancer Res.*, **17**, 365.

Wynder, E. L., Cornfield, J., Schroff, P. D. and Doriswame, K. R.—(1954) *Amer. J. Obstet. Gynec.*, **68**, 1016.